# Construction of Evaluation Indicator System of College Physical Education Teaching Environment Based on Analytic Hierarchy Process

**DOI:** 10.1155/2022/4148866

**Published:** 2022-05-06

**Authors:** Long Zhang

**Affiliations:** Xi'an Physical Education University, Xi'an, Shaanxi 710068, China

## Abstract

As a training base for professional talents, colleges and universities continue to deliver high-quality service-oriented and technical-oriented talents for the country's production, construction, service, and other industries. College students will have to face higher-intensity and higher-density work in their jobs for the future. It requires college students to develop good exercise habits, learn sports knowledge, and build healthy physiques at the university stage so that they can adapt to heavy work in all walks of life. However, on the one hand, there is a relatively lack of research on the systematization of physical education management and the development of physical education resources; on the other hand, physical education in colleges and universities faces problems such as low level of physical education, low utilization of teaching resources, and declining teaching quality. In view of the above problems, this paper proposes the method of literature and materials to carry out the primary selection of the indicators of the evaluation system of physical education teaching environment in colleges and universities. Then, two rounds of screening are carried out for the primary indicators to determine the specific indicators of the evaluation system. Next, the analytic hierarchy process (AHP) is applied to construct the judgment matrix of the indicators at all levels, calculate the weight value of each indicator and do the consistency test, and establish a complete evaluation system of the physical education environment in colleges and universities. Finally, through empirical evidence, it is verified that the constructed evaluation system has operability and feasibility. The evaluation and measurement of colleges and universities carrying out physical education curriculum can ensure the correct and rapid development of physical education teaching and provide reference for the management of physical education resources in colleges and universities.

## 1. Introduction

The development of the teaching environment of physical education in colleges and universities is relatively slow. The results of most studies are that the relevant policies are not perfect, the economic investment in campus football is less, some venues and facilities are lacking, and the football coaches are not very professional. Some national colleges and universities have achieved good results in the physical education environment, but correspondingly, new problems have emerged. The construction of the evaluation indicator system in different regions presents an uneven status quo. The specific performance is that the content of the system is not comprehensive enough, and the selection of indicators is arbitrary [1].

Yin Zhihua mentioned “The environmental framework of physical education includes teaching professional standards, teaching curriculum standards, teaching institution certification standards, and physical education quality evaluation standards. In addition, physical education should adhere to a systematic, scientific, and strict training system, which has the professional and systematic expertise” [2]. In “Development of Educational Resources and Reform of Physical Education in Colleges and Universities,” Liu Zhibin believes that physical education resources are a category with specific connotations. It refers to the value of effectively assisting education and teaching in specific physical education teaching practice and exerting a certain educational function, thereby enhancing students' physique and improving their health. Further, they can master the basic knowledge of sports and enrich cultural life. It can improve the technical level of various sports and is the general term for physical education resources [3]. In “Research on Teaching Resources of Physical Education Courses in Ordinary Colleges,” Xie Jingyue believes that physical education resources are the source of all physical education information, which include all tangible and intangible materials for the development of school sports. Generally speaking, it is divided into six categories: human resources, sports facilities resources, sports project resources, media resources, extracurricular extracurricular resources, and natural environment resources [4]. In “Analysis of Current Situation and Reasonable Allocation of Physical Education Resources in Colleges and Universities,” Shang Jian believes that school physical education resources are various tangible and intangible things, such as strengthening students' physique, expanding physical knowledge, improving health level, and enriching cultural life. It is an umbrella term for the provision of support for physical education and physical activity [5]. Yang Ye pointed out that the professional competence of teachers mainly includes “Physical education teachers' subject knowledge ability, organizational teaching ability, teachers' professional development ability, professional cognitive ability, resource development and utilization ability, ability to recognize students, and ability to evaluate teaching. According to the influence, the above indicators are divided into secondary and tertiary indicators” [6].

Aiming at the problem of insufficient development of physical education resources in colleges and universities, this paper mainly investigates the current situation of teaching resources from multiple dimensions such as curriculum content resources, venue facilities resources, teaching funding resources, and campus cultural resources. In-depth analysis of the survey results, the analytic hierarchy process is used to process the indicator information of the physical education environment. The membership degree of each indicator is evaluated by fuzzy evaluation method, and the indicator weight is established. The research content of this paper will help to improve the level of physical education in colleges and universities, improve the utilization rate of educational resources in colleges and universities, and play a guiding role in the management of college education.

The main innovations of this paper are as follows:

1. Taking the physical education environment of colleges and universities as the investigation object, the analytic hierarchy process is combined with the fuzzy comprehensive evaluation to construct the evaluation model.
2. An indicator system for comprehensive evaluation of the environment of physical education teaching in colleges and universities is built, and a system framework for three-level indicators is also established so that can improve the accuracy of evaluation.

## 2. Related Work

### 2.1. Analytic Hierarchy Process

To apply the analytic hierarchy process, first, the problem should be layered, and the indicators of each layer should be judged. Then, a judgment matrix is formed, and the maximum eigenvalue of the matrix and its corresponding eigenvector are calculated. Next, by classifying the system analysis into the lowest level, the importance weights relative to the highest level indicators are determined. Finally, the weight of this indicator is obtained [7].

1. Building a hierarchy analysis structure. According to the problem and the goal to be achieved, the research indicators are constructed in a hierarchical structure. According to the relationship between the indicators and the relationship between the secondary and tertiary indicators, different levels are combined to form a multilevel analysis structure model.
2. Building a judgment matrix. When the indicator weight is determined, a judgment matrix is required. The relative importance weight is determined by calculating the largest eigenvalue in the judgment matrix and its corresponding eigenvector [8].

### 2.2. Fuzzy Evaluation

Fuzzy comprehensive evaluation is to use the concept of fuzzy mathematics to provide an evaluation method for the actual problem to be solved. In the objective world, there are a lot of ambiguous phenomena. Fuzzy comprehensive evaluation is based on fuzzy mathematics and applies the principle of fuzzy relation synthesis. It is a method to quantify some factors that are not clear in concept and difficult to quantify, and comprehensively evaluate the membership level of the evaluated object from multiple aspects [9].

The set of indicators of the object being evaluated are determined. Next, the weights and membership degree vectors of each indicator are determined, respectively, and the fuzzy judgment matrix is obtained. Finally, the matrix and the weight vector of the indicator are subjected to fuzzy operation and normalization to obtain the fuzzy comprehensive evaluation result. On the basis of constructing the indicator system, this paper continues to advance and explore the steps and methods in the actual operation to prepare for the subsequent empirical studies [10].

To reflect the importance of each indicator, a corresponding weight *a*_*i*_(*i*=1,2,3, ⋯, *m*) is assigned to each indicator *U*, which is usually required as *a*_*i*_ ≥ 0, ∑*a*_*i*_=1. The fuzzy set *A* is formed by the weights of each indicator, which is the weight set. In the process of fuzzy comprehensive evaluation, the weight has a great influence on the final evaluation result. Therefore, the weight calculation method of AHP is adopted [11].

*Synthetic fuzzy comprehensive evaluation result vector*. The different rows in the fuzzy relation matrix *R* reflect the membership degrees of the evaluated object to each level for fuzzy subsets of different indicators. The fuzzy weight vector *A* is used to synthesize different rows, and the membership degree of the evaluation object to each subset is obtained as a whole, that is, the fuzzy comprehensive evaluation result vector *B*, which is *B*=*A*∘*R*.

(1) $$\begin{array}{c} B=A\circ R=\left(a_{1},a_{2},\ldots ,a_{m}\right)\left[\begin{array}{cccc} r_{11} & r_{12} & \ldots & r_{1n}\\ r_{21} & r_{22} & \ldots & r_{2n}\\ \ldots & \ldots & \ldots & \ldots \\ r_{m1} & r_{m2} & \ldots & r_{mn} \end{array}\right]=\left(b_{1},b_{2},\ldots ,b_{n}\right), \end{array}$$

where *b*_*j*_(*j*=1,2,…, *n*) is obtained by the operation of the *j*-th column of *A* and *R*, which can indicate the degree of membership of the rated object to the fuzzy subset of the *A*_*j*_ level as a whole. ∘ is an operator symbol, and *A*∘*R* uses different calculation methods to obtain different result vectors. After analyzing the Delphi method, the weighted average method, and the expert estimation method, it was decided to use the weighted average method for calculation [12]. There are two commonly used fuzzy synthesis operators. Based on the principle of overall indicator, this paper adopts generalized fuzzy operator. If ∑_*i*=1_^*n*^*b*_*j*_ ≠ 1, normalization is required. Given that *b*=*b*_1_+*b*_2_+*b*_3_+⋯+*b*_*n*_=∑_*j*=1_^*n*^*b*_*j*_, it is normalized to

(2) $$\begin{array}{c} B{}^{\prime }=\left(\frac{b_{1}}{b},\frac{b_{2}}{b},\frac{b_{3}}{b},\cdots ,\frac{b_{n}}{b}\right)=\left(b_{1},b_{2},b_{3},\ldots ,b_{n}\right). \end{array}$$

*B*′ is the evaluation result vector of the comprehensive evaluation indicator set *U* for the evaluation set *A*.

## 3. Build an Evaluation Indicator System

**Figure 1:**
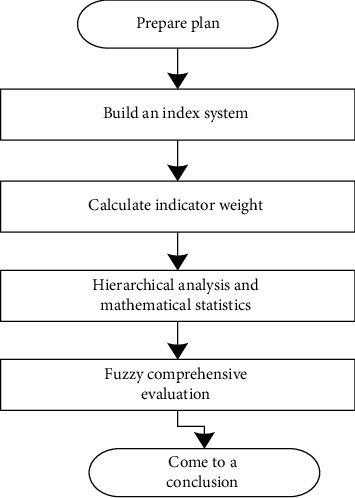
The implementation process of the evaluation system.

It is necessary to understand the content of the evaluation system and the purpose of the relevant influencing factors so that it can lay the foundation for the preliminary establishment of comprehensive evaluation indicators. Then, the indicators at different levels are analyzed and developed. And the corresponding secondary and tertiary indicators are quantified and subdivided to initially form a comprehensive evaluation indicator system. Next, the Delphi method is applied to conduct expert consultation on the initial indicator system. After two rounds, when the opinions of experts converge, the indicator system is completed. Finally, the weights of the indicators are determined according to the calculation, and the indicators are sorted hierarchically to form the final complete and scientific evaluation indicator system. The specific implementation process is shown in Figure 1.

### 3.1. Indicator Screening of Evaluation System

**Figure 2:**
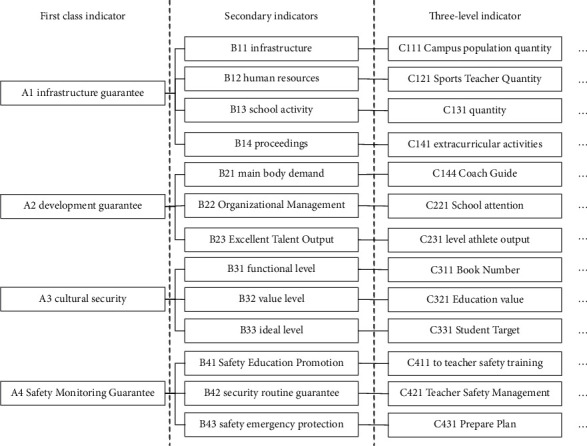
Primary selection indicators for comprehensive evaluation.

A complete system is composed of multiple indicators. Therefore, to determine the subordination of multiple indicators, the indicators can be divided into first-level indicators, second-level indicators, and third-level indicators. In the actual screening process, it is necessary to combine the actual situation to make the indicators scientific, authentic, and operable.  Figure 2 is the primary selection indicators for comprehensive evaluation.

As shown in Figure 2, it includes 4 first-level indicators, 13 second-level indicators, and 41 third-level indicators.

### 3.2. Determining the Evaluation Method

When there are multiple indicators for comprehensive evaluation, the relative weights between the evaluation indicators are different. The more important the index is, the larger the weight coefficient is, and vice versa, the smaller the weight coefficient. Weight is the degree of importance of a factor or indicator to something. Commonly used methods are Delphi method (or expert survey method), subjective experience method, and primary and secondary index queuing classification method. Among the physical education environment indicators in colleges and universities, some indicators cannot be quantified, so the analytic hierarchy process (AHP) method is adopted.

### 3.3. Constructing the Judgment Matrix

The indicator *A* of the upper layer is set as the criterion layer, which is related to the target layer indicator *A*_1_, *A*_2_, *A*_3_, ⋯, *A*_*n*_ of the next layer.  Table 1 is judgment matrix *A* as follows:

(3) $$\begin{array}{c} A=\left[\begin{array}{cccc} 1 & 3 & 1/3 & 1/7\\ 1/5 & 1 & 1/5 & 1/3\\ 3 & 7 & 1 & 1/3\\ 7 & 5 & 3 & 1 \end{array}\right]. \end{array}$$

*A*_*ij*_ is used as the *i*-th metric to compare with the *j*-th metric. When *A*_*ij*_=*K*, *A*_*ji*_=1/*K*.

### 3.4. Scaling Method

Table 2 uses a scaling method of 1–9 and its reciprocal.

The judgment matrix of the above criterion layer *A* to the target layer *A*_*n*_ is shown in Table 3.

The calculation methods and matrix judgments of the second-level and third-level indicators are consistent with the first-level indicators.

### 3.5. Consistency Test of Judgment Matrix

To make the experts' judgments of the importance of the indicators consistent, and there is no contradiction, the consistency test of the matrix should be carried out.

According to the relevant theory of the matrix, if *λ* satisfies *Ax*=*λx*, then *λ* is the feature value of A. For *A*_*ij*_=1, then

(4) $$\begin{array}{c} \sum_{i=1}^{n}\lambda_{i}=n. \end{array}$$

Obviously, when the matrix is completely consistent, then *λ*_1_=*λ*_max_=*n*, and the rest of the eigenvalue roots are all 0. And when the matrix is not completely consistent, then *λ*_1_=*λ*_max_ > *n*, and the rest of the root *λ*_2_, *λ*_3_,…, *λ*_n_ have the following relationship:

(5) $$\begin{array}{c} \sum_{i=2}^{n}\lambda_{i}=n-\lambda_{\max}. \end{array}$$

Therefore, in the judgment matrix, the indicators in the physical education environment system of colleges and universities are subjective in the same set. When the unit eigenvector is not credible, the negative mean of the remaining eigenvalues in the matrix will be introduced to calculate the consistency index, and the formula is as follows:

(6) $$\begin{array}{c} CI=\frac{\lambda_{\max}-n}{n-1}. \end{array}$$

The larger the *CI* value, the greater the deviation consistency of the judgment matrix; the smaller the *CI* value (closer to 0), the better the consistency of the judgment matrix. When *CI*=0, the judgment matrix is completely consistent. When the judgment matrix has satisfactory consistency, the average random consistency index needs to be introduced.  Table 4 is the value for the judgment matrix of 1–9 order.

When the order is greater than 2, the ratio of the judgment matrix consistency indicator *CI* and the random consistency index *RI* becomes the random consistency ratio *CR*. When *CR*=*CI*/*RI* < 0.01, it can be shown that the matrix has satisfactory consistency; otherwise, it needs to be readjusted.

### 3.6. Hierarchical Ordering

The calculation of hierarchical single ordering is the problem of calculating the largest eigenroot and eigenvector in the judgment matrix. Because this calculation has considerable error, it is not required to be the most precise. Its calculation steps are as follows:

(7) $$\begin{array}{c} M_{i}=\prod_{j=1}^{n}a_{ij}. \end{array}$$

(1) The product *Mi* of the elements of each row in the judgment matrix is calculated. It is calculated according to the follow equation:
  (8) $$\begin{array}{c} M1=1\times 3\times \frac{1}{3}\times \frac{1}{7}=\frac{1}{7},\\ M2=\frac{1}{5}\times 1\times \frac{1}{5}\times \frac{1}{3}=\frac{1}{75},\\ M3=3\times 7\times 1\times \frac{1}{3}=7,\\ M4=7\times 5\times 3\times 1=105. \end{array}$$
(2) To open the *n*-th root $\bar{W_{i}}$ of *M*_*i*_ of *B*_1_, *B*_2_, and *B*_3_, the system constructed this time has 4 first-level indicators, so *n*=4 is substituted into following equations:
  (9) $$\begin{array}{c} \bar{W_{i}}=\sqrt[{n}]{Mi}, \end{array}$$
  (10) $$\begin{array}{c} \bar{W_{1}}=\sqrt[{4}]{\frac{1}{7}}=0.6148,\\ \bar{W_{3}}=\sqrt[{4}]{7}=1.6266,\\ \bar{W_{4}}=\sqrt[{4}]{105}=3.2011. \end{array}$$
(3) According to equation (11), the vector $\bar{W}=\left[\bar{W_{1}},\bar{W_{2}},\bar{W_{3}},\bar{W_{4}}\right]$ is normalized to obtain the weight feature vector *W*=[*W*_1_, *W*_2_, *W*_3_, *W*_4_].
  (11) $$\begin{array}{c} W_{i}=\frac{\bar{W_{i}}}{\sum_{j=1}^{n}\bar{W_{j}}}, \end{array}$$
  (12) $$\begin{array}{c} W_{1}=\frac{0.6148}{\left(0.6148+0.3398+1.6266+3.2011\right)}=0.1063,\\ W_{3}=\frac{1.6266}{\left(0.6148+0.3398+1.6266+3.2011\right)}=0.2813,\\ W_{4}=\frac{3.2011}{\left(0.6148+0.3398+1.6266+3.2011\right)}=0.5536. \end{array}$$
(4) The largest eigenvalue *λ*_max_ in the judgment matrix is calculated, as shown in expression (12), where (*AW*)*i* represents the *i*-th element of the vector *AW*. The obtained *A*_*ij*_ and *W*_*i*_ are substituted into equation (14)as follows:
  (13) $$\begin{array}{c} \left(AW\right)i=A_{ij}\times W_{i}, \end{array}$$
  (14) $$\begin{array}{c} AW=\left[\begin{array}{cccc} 1 & 3 & \frac{1}{3} & \frac{1}{7}\\ \frac{1}{5} & 1 & \frac{1}{5} & \frac{1}{3}\\ 3 & 7 & 1 & \frac{1}{3}\\ 7 & 5 & 3 & 1 \end{array}\right]\times \left[\begin{array}{c} 0.1063\\ 0.0588\\ 0.2813\\ 0.5536 \end{array}\right]. \end{array}$$
  and obtain
  (15) $$\begin{array}{c} \left(AW\right)1=1\times 0.1063+3\times 0.0588+\frac{1}{3}\times 0.2813+\frac{1}{7}\times 0.5536=0.4556,\\ \left(AW\right)2=\frac{1}{5}\times 0.1063+1\times 0.0588+\frac{1}{5}\times 0.2813+\frac{1}{3}\times 0.5536=0.3208,\\ \left(AW\right)3=3\times 0.1063+7\times 0.0588+1\times 0.2813+\frac{1}{3}\times 0.5536=1.1963,\\ \left(AW\right)4=7\times 0.1063+5\times 0.0588+3\times 0.2813+1\times 0.5536=1.6163. \end{array}$$
  (*AW*)*i*={(*AW*)1, (*AW*)2, (*AW*)3, (*AW*)4} is substituted into following equation.
  (16) $$\begin{array}{c} \lambda_{\max}=\sum_{i=1}^{n}\frac{{\left(AW\right)}_{i}}{nW_{i}}. \end{array}$$
  It is given that *λ*_max_=1/4 × (0.4556/0.1063+0.3208/0.0588+1.1963/0.2813+1.6163/0.5536)=4.2285.
(5) Determining whether the matrix is consistent by calculating the consistency test index. The calculated *λ*_max_ is substituted into equation (17). There are 4 first-level indicators, so *n*=4.
  (17) $$\begin{array}{c} CI=\frac{\lambda_{\max}-n}{n-1}, \end{array}$$
  (18) $$\begin{array}{c} CI=\frac{4.2285-4}{3}=0.0762. \end{array}$$

According to the *RI* value table of the broken matrix, when the order is 4, *RI*=0.90 is substituted into equation (19), and *CR*=0.0847 is calculated. Due to *CR* < 0.1, it can be judged that the first-level index matrix has satisfactory consistency.

(19) $$\begin{array}{c} CR=\frac{CI}{RI}. \end{array}$$

## 4. Application of Fuzzy Comprehensive Evaluation

### 4.1. Single-Factor Evaluation and Establishment of Fuzzy Relationship Matrix

Evaluation is performed from a single indicator to determine the membership degree of the evaluation object to the evaluation set *W*, which is called single-indicator fuzzy evaluation. After constructing the hierarchical fuzzy subset, the evaluation objects should be quantified one by one from each indicator *u*_*i*_(*i*=1,2,3, ⋯, *m*); that is, the membership degree of the evaluation object to each level of fuzzy subsets is determined from a single indicator so that can obtain the fuzzy relationship matrix.

(20) $$\begin{array}{c} R=\left(\begin{array}{cccc} r_{11} & r_{12} & \cdots & r_{1n}\\ r_{21} & r_{22} & \cdots & r_{2n}\\ \cdots & \cdots & \cdots & \cdots \\ r_{m1} & r_{m2} & \cdots & r_{mn} \end{array}\right), \end{array}$$

where *r*_*ij*_(*i*=1,2, ⋯, *m*; *j*=1,2, ⋯, *n*) represents the membership degree of the fuzzy subset of *W*_*j*_ level viewed from the *u*_*i*_ of an evaluated object. The performance of the evaluation object in some indicators *u*_*i*_ is characterized by the fuzzy vector *r*_*i*_=(*r*_*i*1_, *r*_*i*2_, ⋯*r*_*im*_). *r*_*i*_ is called a single index evaluation matrix, which can be regarded as the fuzzy relationship between the factor set *U* and the evaluation set *W*.

When determining affiliation, the grades and scores are usually evaluated by experts and teachers or related professionals. Then, the statistical score result *r*_*ij*_ is obtained according to the absolute value subtraction, namely,

(21) $$\begin{array}{c} r_{ij}=\left\{\begin{array}{c} 1,\left(i=j\right)\\ 1-c\sum_{k=1}\left|x_{ik}-x_{jk}\right|,\left(i\neq j\right) \end{array}\right), \end{array}$$

where *c* can be appropriately selected such that 0 ≤ *r*_*ij*_ ≤ 1.

### 4.2. Analysis of Fuzzy Comprehensive Evaluation Results

The result of fuzzy comprehensive evaluation is the membership degree of the evaluation object to each level of fuzzy subsets. Because it is a fuzzy vector rather than a point value, it needs to handle comparisons of multiple evaluation objects. The comprehensive score of each evaluation object is calculated, and the best evaluation object is selected in order. The processing methods include the principle of maximum membership degree and the principle of weighted average. In some cases, the application of the maximum membership principle may be far-fetched, which may not only result in the loss of more information but also lead to unreasonable evaluation results. Therefore, the weighted average principle will be adopted in this paper.

The principle of weighted average is to take the rank as a relative position and make it continuous. In order to be quantifiable, the grades will be represented by “1, 2, 3,…, *m.*” The sum of each level is weighted with the corresponding components in *B* so that it can obtain the relative position of the evaluated object (22) is shown as follows.

(22) $$\begin{array}{c} A=\frac{\sum_{j=1}^{n}b_{j}^{k}\cdot j}{\sum_{j=1}^{n}b_{j}^{k}}, \end{array}$$

where *k* is the undetermined coefficient (*k* = 1 or 2), its purpose is to control the effect caused by the larger *b*_*j*_. When *K*⟶*∞*, the weighted average principle is the maximum membership principle.

## 5. Application of Fuzzy Comprehensive Evaluation

### 5.1. Determining the Comprehensive Evaluation Judgment Set

Determining the comprehensive evaluation judgment set plays an important role in the fuzzy comprehensive evaluation. The rational, scientific, and standardized evaluation process will directly affect whether the evaluation results are correct. The construction of the physical education environment index system in colleges and universities has been completed, and the weight coefficients of each index have also been calculated. To further screen the universe of comprehensive evaluation factors, this paper uses the Likert 5-level scoring method to qualitatively evaluate the indicator evaluation vocabulary *W* = {excellent, good, average, poor, poorer}, and the corresponding evaluation values are {1.0, 0.8, 0.6, 0.4, 0.2}.

### 5.2. Determining the Fuzzy Comprehensive Evaluation Factor Set

According to the construction of the physical education environment index system in colleges and universities, the factor set is divided into three levels, which are the first-level index factor layer: *U*={*A*_1_, *A*_2_, *A*_3_, *A*_4_}; the second-level index factor layer: *A*_1_=[*B*_11_, *B*_12_, *B*_13_, *B*_14_], *A*_2_=[*B*_21_, *B*_22_, *B*_23_], *A*_3_=[*B*_31_, *B*_32_, *B*33], and *A*_4_=[*B*_41_, *B*_42_, *B*_43_]; the third-level index factor layer: *B*_11_=[*C*_111_, *C*_112_, *C*_113_], *B*_12_=[*C*_121_, *C*_122_, *C*_123_, *C*_124_], *B*_13_=[*C*_131_, *C*_132_, *C*_133_], *B*_14_=[*C*_141_, *C*_142_, *C*_143_, *C*_144_], *B*_21_=[*C*_211_, *C*_212_], *B*_22_=[*C*_221_, *C*_222_, *C*_223_], *B*_23_=[*C*_231_, *C*_232_, *C*_233_], *B*_31_=[*C*_311_, *C*_312_, *C*_313_, *C*_314_], *B*_32_=[*C*_321_, *C*_322_, *C*_323_], *B*_33_=[*C*_331_, *C*_332_, *C*_333_], *B*_41_=[*C*_411_, *C*_412_], *B*_42_=[*C*_421_, *C*_422_, *C*_423_], and *B*_43_=[*C*_431_, *C*_432_, *C*_433_].

### 5.3. Calculation of Each Score of Evaluation Indicator

There are 4 first-level indicators and 13 second-level indicators to construct the physical education environment index system in colleges and universities, and each first-level index contains several second-level indicators. They are calculated and analyzed separately, and then, 47 expert indicators are evaluated for statistics. Taking the basic system guarantee as an example, the calculation process is described in detail.

(1) Establishment of a fuzzy relationship matrix
  (23) $$\begin{array}{c} R11=\left[\begin{array}{ccccc} \text{0.}064 & \text{0.}362 & \text{0.}383 & \text{0.}149 & \text{0.}043\\ \text{0.}043 & \text{0.}234 & \text{0.}277 & \text{0.}298 & \text{0.}149\\ 0.319 & 0.255 & 0.234 & 0.128 & 0.064 \end{array}\right]\frac{1}{2},\\ R12=\left[\begin{array}{c} \begin{array}{ccccc} \text{0.}170 & \text{0.}319 & \text{0.}362 & \text{0.}106 & \text{0.}043\\ \text{0.}213 & \text{0.}340 & \text{0.}298 & \text{0.}149 & \text{0.}000\\ 0.085 & 0.170 & 0.404 & 0.319 & 0.021 \end{array}\\ \begin{array}{ccccc} 0.149 & 0.383 & 0.298 & 0.085 & 0.085 \end{array} \end{array}\right],\\ R13=\left[\begin{array}{ccccc} \text{0.}128 & \text{0.}362 & \text{0.}340 & \text{0.}149 & \text{0.}021\\ \text{0.}043 & \text{0.}234 & \text{0.}362 & \text{0.}255 & \text{0.}106\\ 0.255 & 0.340 & 0.319 & 0.043 & 0.043 \end{array}\right],\\ R14=\left[\begin{array}{c} \begin{array}{ccccc} \text{0.}043 & \text{0.}191 & \text{0.}447 & \text{0.}234 & \text{0.}085\\ \text{0.}574 & \text{0.}277 & \text{0.}106 & \text{0.}043 & \text{0.}000\\ 0.021 & 0.064 & 0.383 & 0.404 & 0.128 \end{array}\\ \begin{array}{ccccc} 0.298 & 0.255 & 0.277 & 0.128 & 0.043 \end{array} \end{array}\right]. \end{array}$$
(2) The fuzzy comprehensive evaluation result vector is synthesized. The fuzzy weight vector A is used to synthesize different rows to obtain the overall membership degree of the evaluation object to each subset, that is, the fuzzy comprehensive evaluation result vector *B*, which is *B*=*A*∘*R*. The weight coefficient of each index is determined, so the index factor evaluation score of the secondary index of the basic guarantee system can be calculated as follows:
  (24) $$\begin{array}{c} B11=R11\circ A11=\left[\begin{array}{ccccc} \text{0.}064 & \text{0.}362 & \text{0.}383 & \text{0.}149 & \text{0.}043\\ \text{0.}043 & \text{0.}234 & \text{0.}277 & \text{0.}298 & \text{0.}149\\ 0.319 & 0.255 & 0.234 & 0.128 & 0.064 \end{array}\right]\circ \left[\begin{array}{ccc} 0.5272 & 0.4038 & 0.4583 \end{array}\right]\\ =\left[\begin{array}{ccccc} \text{0.}1973019 & \text{0.}4022021 & \text{0.}4210124 & \text{0.}2575476 & \text{0.}112167 \end{array}\right],\\ B12=R12\circ A12=\left[\begin{array}{c} \begin{array}{ccccc} \text{0.}170 & \text{0.}319 & \text{0.}362 & \text{0.}106 & \text{0.}043\\ \text{0.}213 & \text{0.}340 & \text{0.}298 & \text{0.}149 & \text{0.}000\\ 0.085 & 0.170 & 0.404 & 0.319 & 0.021 \end{array}\\ \begin{array}{ccccc} 0.149 & 0.383 & 0.298 & 0.085 & 0.085 \end{array} \end{array}\right]\circ \left[\begin{array}{ccc} 0.4301 & 0.4158 & 0.3902 \end{array}\;0.4478\right]\\ =\left[\begin{array}{ccccc} \text{0.}2615716 & \text{0.}5164153 & \text{0.}5706898 & \text{0.}2700816 & \text{0.}0647515 \end{array}\right],\\ B13=R13\circ A13=\left[\begin{array}{ccccc} \text{0.}128 & \text{0.}362 & \text{0.}340 & \text{0.}149 & \text{0.}021\\ \text{0.}043 & \text{0.}234 & \text{0.}362 & \text{0.}255 & \text{0.}106\\ 0.255 & 0.340 & 0.319 & 0.043 & 0.043 \end{array}\right]\circ \left[\begin{array}{ccc} 0.3897 & 0.4722 & 0.5124 \end{array}\right]\\ =\left[\begin{array}{ccccc} \text{0.}2008482 & \text{0.}4257822 & \text{0.}4668900 & \text{0.}2005095 & \text{0.}0802701 \end{array}\right],\\ B14=R14\circ A14=\left[\begin{array}{c} \begin{array}{ccccc} \text{0.}043 & \text{0.}191 & \text{0.}447 & \text{0.}234 & \text{0.}085\\ \text{0.}574 & \text{0.}277 & \text{0.}106 & \text{0.}043 & \text{0.}000\\ 0.021 & 0.064 & 0.383 & 0.404 & 0.128 \end{array}\\ \begin{array}{ccccc} 0.298 & 0.255 & 0.277 & 0.128 & 0.043 \end{array} \end{array}\right]\circ \left[\begin{array}{ccc} 0.6718 & 0.3728 & 0.6654\text{ 0.}3942 \end{array}\right]\\ =\left[\begin{array}{ccccc} \text{0.}3743196 & \text{0.}3746860 & \text{0.}7038530 & \text{0.}4925108 & \text{0.}1592248 \end{array}\right],\\ B1=R1\circ A1=\left[\begin{array}{ccccc} \text{0.}1973019 & \text{0.}4022021 & \text{0.}4210124 & \text{0.}2575476 & \text{0.}1121670\\ \text{0.}2615716 & \text{0.}5164153 & \text{0.}5706898 & \text{0.}2700816 & \text{0.}0647515\\ \text{0.}2008482 & \text{0.}4257822 & \text{0.}4668900 & \text{0.}2005095 & \text{0.}0802701\\ \text{0.}3743196 & \text{0.}3746860 & \text{0.}7038530 & \text{0.}4925108 & \text{0.}1592248 \end{array}\right]\circ \left[\begin{array}{cccc} \text{0.}5036 & \text{0.}5107 & \text{0.}3894 & \text{0.}3405 \end{array}\right]\\ =\left[\begin{array}{ccccc} \text{0.}43861196584 & \text{0.}75966244295 & \text{0.}424942038 & \text{0.}51402812436 & \text{0.}17502911359 \end{array}\right]. \end{array}$$
(3) According to the results of the three-level index fuzzy comprehensive evaluation in the last two steps, construct a basic system guarantee two-level index fuzzy membership degree relation matrix and establish a fuzzy comprehensive model of the basic system guarantee two-level index by combining the weight coefficients of the two-level indexes: *B1* is normalized to *A*1′=[0.219305983  0.379831221  0.212471019  0.125877227  0.06251455] due to ∑_*j*=1_^*n*^*b*_*j*_ ≠ 1. The basic system assurance evaluation score is
  (25) $$\begin{array}{c} A1^{\prime }\times W=\left[\begin{array}{ccccc} \text{0.}219305983 & \text{0.}379831221 & \text{0.}212471019 & \text{0.}125877227 & \text{0.}06251455 \end{array}\right]\times \left[\begin{array}{c} 1\\ 0.8\\ 0.6\\ 0.4\\ 0.2 \end{array}\right]=\text{0.}713507372 \end{array}$$

Finally, the comprehensive evaluation index score of the basic system guarantee obtained is 0.713507372, which is in a good grade. The data calculated above have been normalized, so the score of the three-level indicator can be directly calculated as

(26) $$\begin{array}{c} A=\left[\begin{array}{cccc} \text{0.}713507372 & \text{0.}80547233 & \text{0.}643415834 & \text{0.}81493407 \end{array}\right]\times \left[\begin{array}{c} \text{0.}1063\\ \text{0.}0588\\ \text{0.}2813\\ \text{0.}5536 \end{array}\right]=\text{0.}75534798 \end{array}$$

After calculation, it can be obtained that the comprehensive evaluation score of physical education teaching environment in colleges and universities is 0.75534798, which is between “good” and “average.” According to the score table, it can be determined that the comprehensive evaluation level of the physical education teaching environment in colleges and universities is in the “good” level.

### 5.4. Comparative Analysis of Algorithm Performance

In this paper, the comprehensive evaluation based on the analytic hierarchy process is compared with the traditional expert evaluation and the simple index evaluation. In view of the teaching environment of physical education in colleges and universities, the calculation accuracy is compared and analyzed.

The expert analysis method requires higher personal ability of experts, and it is easy to produce nonobjective form, and there are more uncertainties; while the simple index evaluation is the key to establish the weight quality of the index, it is difficult to fully reflect the complete reflection of the educational environment.

The comparison matrix is shown in Table 5.

**Figure 3:**
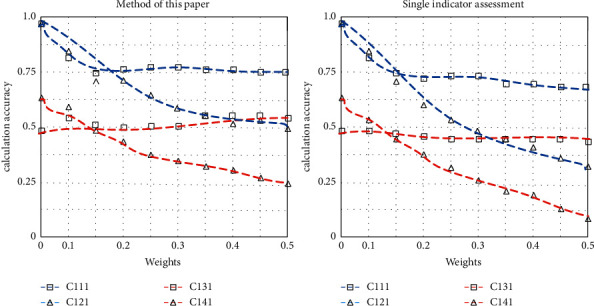
Comparison of evaluation tests.

Because the evaluation values obtained by expert analysis are quite different, they cannot represent the rationality of the index value. The effect of single index evaluation is close to that of comprehensive evaluation in this paper, but the accuracy is still greater than that of the method in this paper. The results are shown in Figure 3.

The maximum eigenvalue and the eigenvector of the matrix C are obtained by using the function. The CR value is less than 0.1 through calculation so that the judgment matrix meets the consistency requirement and can be used for calculating the weight vector. The results show that the calculation accuracy of the comprehensive evaluation in this paper is higher than that of the single index evaluation.

## 6. Conclusion

This paper is committed to building a comprehensive evaluation system, which is in line with the evaluation index system of the physical education teaching environment in colleges and universities. It is used to locate the physical education environment in colleges and universities, clarify the development direction, cultivate a standardized physical education environment, and cultivate the output of excellent talents in colleges and universities, and correctly guide the value and connotation of the physical education environment in colleges and universities. The main work of this paper is as follows:

1. The comprehensive evaluation system of physical education teaching environment in colleges and universities is constructed for multi-index evaluation.
2. The weight of the comprehensive evaluation index system of physical education teaching environment in colleges and universities is established by using the analytic hierarchy process.
3. Fuzzy comprehensive evaluation method is used to evaluate the physical education environment in colleges and universities, so that it can obtain the scores.

The empirical part of this paper only selects the questionnaire survey, data statistics, and score calculation. In the future research, more characteristic schools will be selected for horizontal comparison, and a more complete, scientific, and maneuverable index system for comprehensive evaluation of the physical education environment in colleges and universities will be constructed.

## Figures and Tables

**Table 1:** General form of judgment matrix.

*A*	*A*_*1*_	…	*A*_*n*_
*A*_1_	*A*_11_	…	*A*_1*n*_
*A*_2_	*A*_21_	…	*A*_2*n*_
*A*_*n*_	*A*_*n*1_	…	*A*_*nn*_

**Table 2:** Scaling method.

No	Importance level	*A*_*ij*_ assignment
1	*i* and *j* two elements are equally important	1
2	*i* element is slightly more important than the *j* element	3
3	*i* element is significantly more important than the *j* element	5
4	*i* element is strongly more important than the *j* element	7
5	*i* element is extremely important than the *j* element	9
6	*i* elements are slightly less important than *j* elements	1/3
7	*i* elements are significantly less important than *j* elements	1/5
8	*i* element is strongly less important than the *j* element	1/7
9	*i* elements are extremely less important than *j* elements	1/9

**Table 3:** Judgment matrix of first-level indicators.

A	*A*_1_	*A*_2_	*A*_3_	*A*_4_
*A*_1_	1	3	1/3	1/7
*A*_2_	1/5	1	1/5	1/3
*A*_3_	3	7	1	1/3
*A*_4_	7	6	3	1

**Table 4:** RI value table of order judgment matrix.

Order	1	2	3	4	5	6	7	8	9
RI value	0.00	0.00	0.58	0.90	1.12	1.24	1.32	1.41	1.45

**Table 5:** Evaluation comparison matrix.

Comparison indicators	Weight value			Evaluation results		
	Expert analysis	Indicator evaluation	Comprehensive evaluation	Expert analysis	Indicator evaluation	Comprehensive evaluation
C111	4.64	0.457	0.099	3.18	0.515	0.162
C121	4.27	0.641	0.150	3.73	0.455	0.122
C131	3.91	0.654	0.167	3.54	0.612	0.173
C141	3.82	0.366	0.096	3.82	0.778	0.204

## Data Availability

The experimental data used to support the findings of this study are available from the corresponding author upon request.
